# Supplementary material for: Control of MXenes’ electronic properties through termination and intercalation
Source: Nat Commun. 2019 Jan 31;10:522. doi: 10.1038/s41467-018-08169-8 (PMC6355901; doi:10.1038/s41467-018-08169-8)
Supplement: Supplementary file 1 — Supplementary Information [file 41467_2018_8169_MOESM1_ESM.pdf]

Supplementary Information

Control of MXenes' Electronic Properties Through Termination and Intercalation

Hart et al.

Supplementary Figures

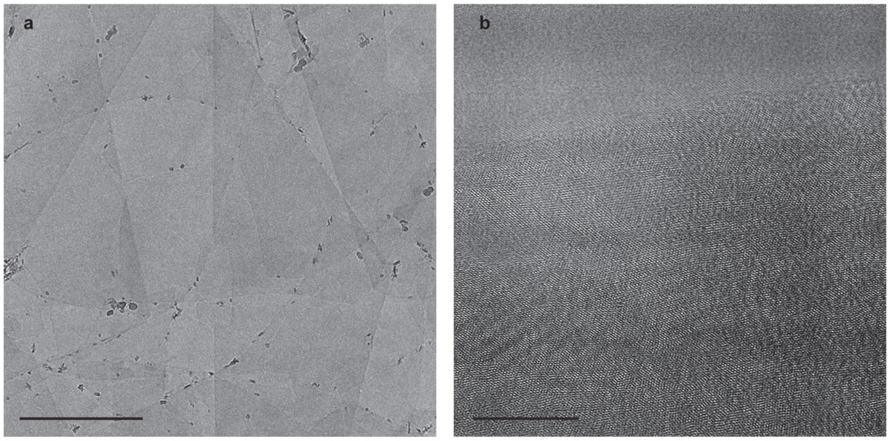

Supplementary Figure 1 MXene stability with electron irradiation and annealing. Low magnification **(a)** and high magnification **(b)** TEM images of overlapping $Ti_3C_2T_x$ flakes after annealing at >500 °C within the TEM and exposure to electron irradiation for imaging, EELS, and diffraction. For **a**, the scale bar is 500 nm, and for **b**, the scale bar is 10 nm. While there is some TiO_2 formation seen in **a**, there are no voids present in the MXene flakes, in stark contrast to recent reports by Sang *et al*[1]. TEM imaging shows that with low current (and low current density) imaging and spectroscopy, there is minimal electron beam induced sample degradation. This claim is further supported by the retention of –O terminations after annealing $Ti_3C_2T_x$ at 775 °C (see Fig. 3c in the main text).

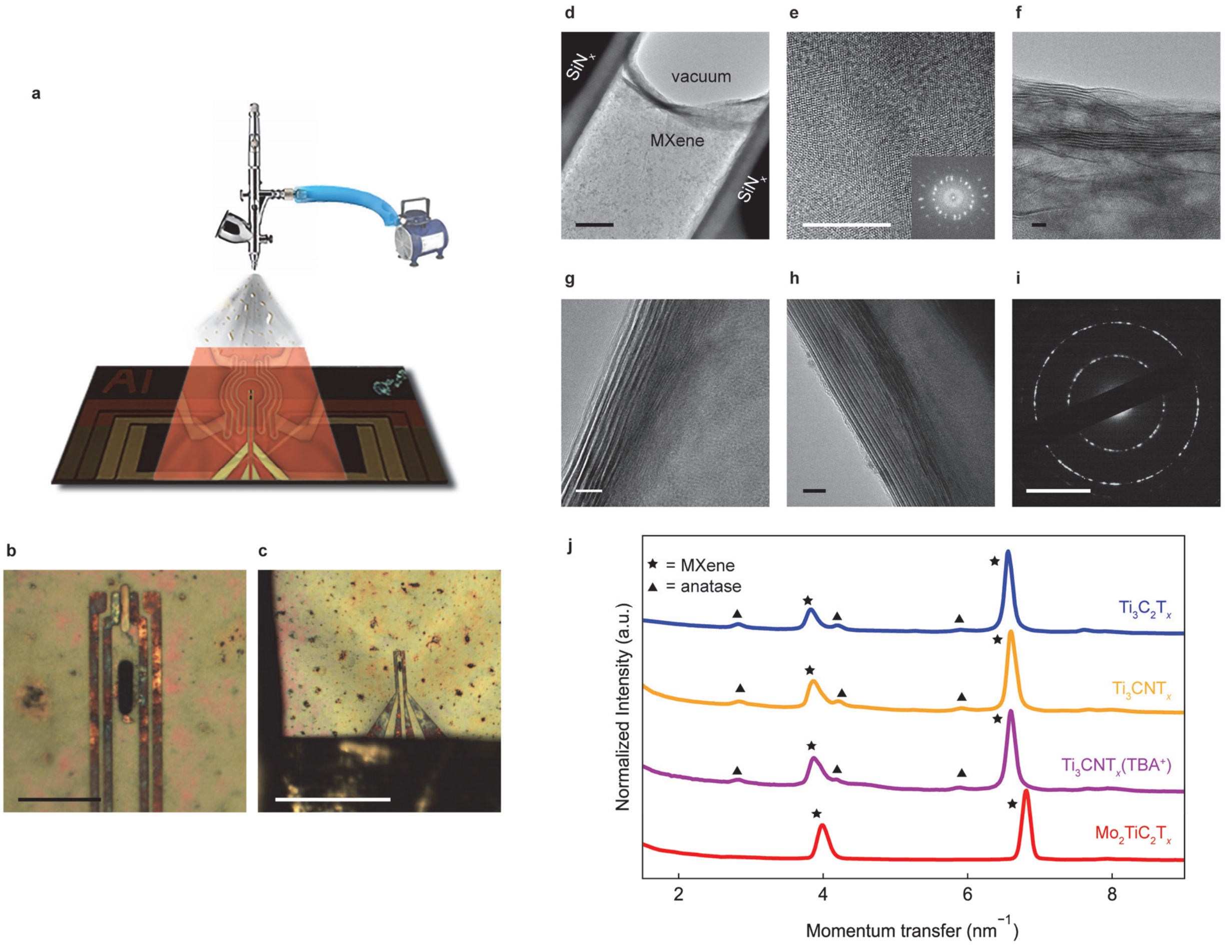

Supplementary Figure 2 MXene device fabrication and characterization. **a,** Schematic showing the heating and biasing nanochip and the spray casting technique. The central region on the nanochip is a thin SiN_x window, and this area is enlarged in the schematic for clarity. The SiN_x window contains circular heating coils that allow *in situ* (resistive) heating up to 800 °C. Additionally, there are 4 Pt electrodes for 4-terminal electrical measurements. Between the middle two Pt electrodes, there are two open windows for TEM imaging. The nanochip fits into an *in situ* TEM sample holder, which is then inserted in the TEM. An external computer controls the heating and biasing. **b,** Optical image of the biasing electrodes of the nanochip after spray casting of $Ti_3C_2T_x$, scale bar = 30 µm. The deposited MXene film spans the Pt electrodes and fully bridges one of the windows for TEM viewing (top window). **c,** Optical image showing the mask used to confine the MXene to the area above the electrodes, scale bar = 175 µm. For **b** and **c**, images show a biasing only nanochip, not a heating and biasing nanochip. **d,** Low magnification TEM image of a $Ti_3C_2T_x$ MXene film deposited onto a nanochip, scale bar = 1 µm. The image spans the width of the viewing window, showing the SiN_x membrane of the nanochip. For this sample, the MXene film only spanned a portion of the viewing window. **e-h**, High resolution TEM images of the four MXene samples studied here: $Ti_3C_2T_x$ **(e)**, Ti_3CNT_x **(f)**, $Ti_3CNT_x(TBA^+)$ **(g)**, and $Mo_2TiC_2T_x$ **(h)**. In each panel, the scale bar corresponds to 5 nm. In **e**, the $Ti_3C_2T_x$ sample is imaged with the electron beam parallel to the MXene *c*-axis. The inset shows the image Fourier transform, with the inner and outer rings corresponding to $(1\bar{1}00)$ and $(11\bar{2}0)$ planes, respectively. In **f-h**, the MXenes are imaged edge on, perpendicular to the *c*-axis. Their layered structure is evident. **i,** Selected area electron diffraction (SAED) pattern of as-prepared $Ti_3C_2T_x$. The scale bar is 5 nm^{-1}. Similar to the Fourier transform in **e**, the inner and outer rings correspond to $(1\bar{1}00)$ and $(11\bar{2}0)$ reflections, respectively. **j,** Rotationally averaged (RA)-SAED patterns of all MXene samples after final annealing at ≥700 ºC. While small amounts of anatase are present in $Ti_3C_2T_x$ and Ti_3CNT_x after annealing[2], the RA-SAED data demonstrates that the MXene samples largely retained their structure during high temperature annealing.

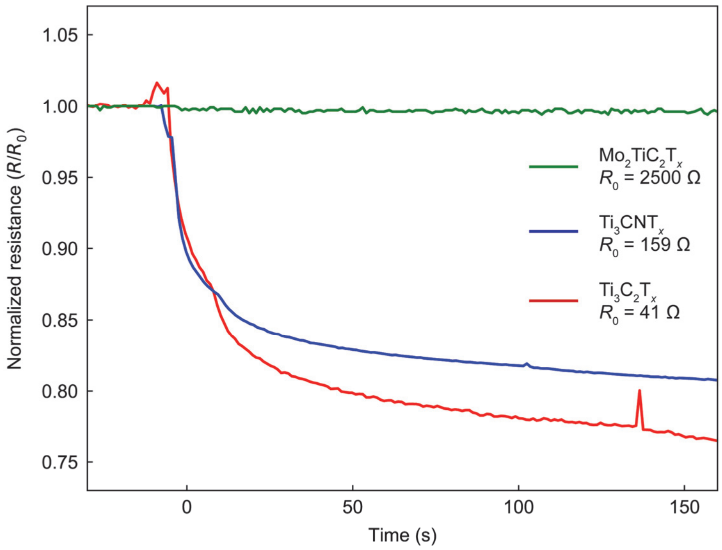

Supplementary Figure 3 Effect of adsorbed species on resistance. Normalized resistance of the $Ti_3C_2T_x$, Ti_3CNT_x, and $Mo_2TiC_2T_x$ MXene films upon initial insertion into the TEM. R_0 is the resistance at time = 0 s, right before the sample was inserted into the TEM vacuum. The decrease in sample resistance is attributed to the loss of adsorbed species, e.g. H_2O and O_2. The $Ti_3CNT_x(TBA^+)$ sample is not shown, since initial resistance values were so high that they could not accurately be determined with the experimental set-up which limited the applied bias to 5 mV.

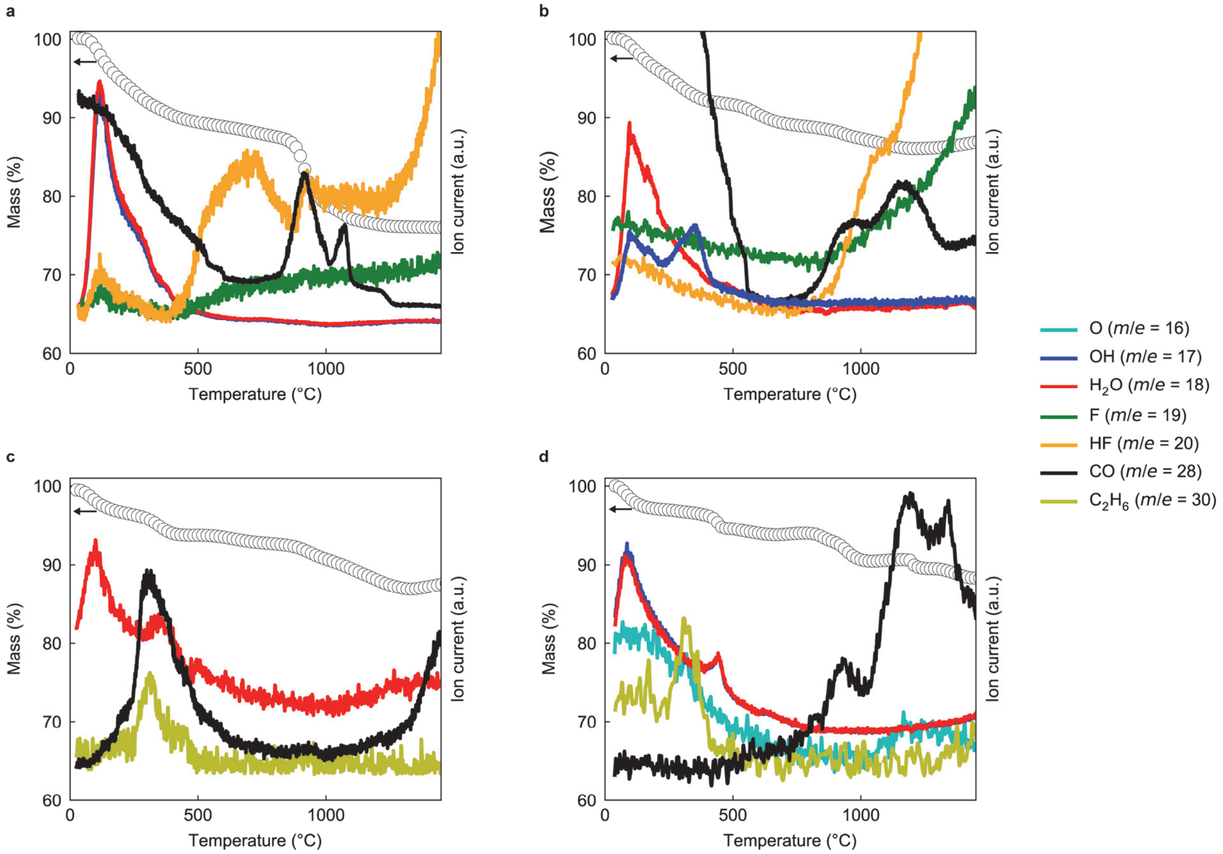

Supplementary Figure 4 TGA-MS data of all studied MXenes. Results for $Ti_3C_2T_x$ **(a)**, Ti_3CNT_x **(b)**, $Ti_3CNT_x(TBA^+)$ **(c)**, and $Mo_2TiC_2T_x$ **(d)** are shown. In all cases, a peak in the H_2O ion current at ~150 °C indicates the release of H_2O intercalants. In most cases, the –OH signal mirrors the H_2O signal, indicating that de-protonated H_2O dominates the measured –OH ion channel. However, the –OH signal from Ti_3CNT_x (see panel **b**) shows a clear peak at ~375 °C which is distinct from the measured H_2O ion current, indicating the release of –OH surface groups. In **a** and **b**, the –F and HF ion currents indicate the loss of –F surface terminations from $Ti_3C_2T_x$ and Ti_3CNT_x, beginning as low as ~400 °C. The broad peaks centered at 150 °C in the –F and HF signals perfectly match that of the H_2O channel, indicating that these peaks are related to H_2O loss. In **c** and **d**, the loss of TBA^+ from $Ti_3CNT_x(TBA^+)$ and $Mo_2TiC_2T_x$ is indicated by the peak in the C_2H_6 (*m/e* = 30) ion current. In **d**, a clear signal in the –O ion channel (*m/e* = 16) is observed beginning at ~1000 °C, suggesting loss of –O from $Mo_2TiC_2T_x$. For the Ti based MXenes, no loss of –O was observed with TGA-MS up to 1500 °C. In all cases, the drop in mass % and the spike in the CO ion current at temperatures above ~800 °C indicate the onset of MXene decomposition.

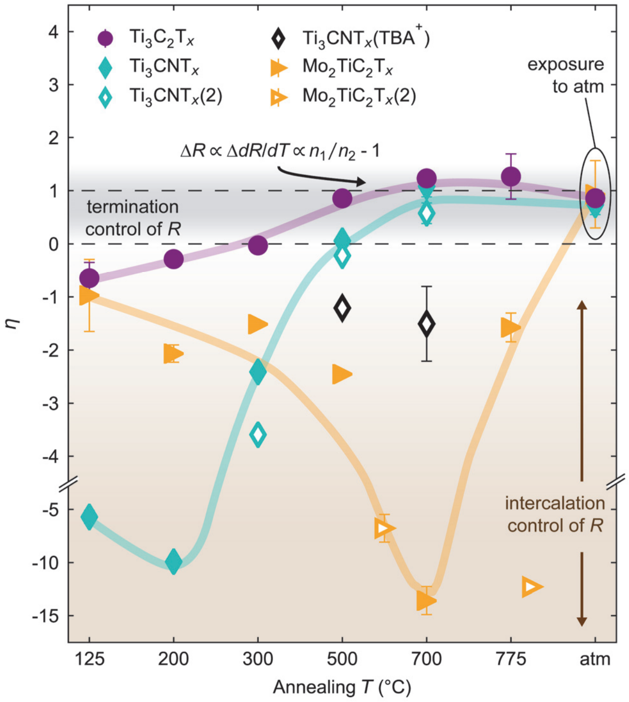

Supplementary Figure 5 Analysis of concurrent resistance and *dR*/*dT* changes with annealing. Plot of η for each annealing step. For a given annealing step, η is the ratio of the proportional change in *dR*/*dT* to the proportional change in resistance (Supplementary Equation 21). A positive value of η is consistent with a change in the intra-flake resistance, and a negative value of η is consistent with a change to the inter-flake resistance. For a change in resistance solely due to a change in the intra-flake carrier concentration, the Drude formula predicts that the value of $\eta = 1$. The solid markers correspond to the $Ti_3C_2T_x$, Ti_3CNT_x, and $Mo_2TiC_2T_x$ samples shown in Fig. 1 of the main text. The colored lines are a guide to the eye for these samples. The open markers represent MXene samples not shown in Fig. 1. The $Ti_3CNT_x(TBA^+)$ sample was annealed at 500 and 700 °C, the $Ti_3CNT_x(2)$ sample was annealed at 300, 500 and 700 °C, and the $Mo_2TiC_2T_x(2)$ sample was annealed at 600 and 790 °C (see Supplementary Tables 1 and 2). Error bars represent the measurement standard deviation accounting for the linear fit to the *dR*/*dT* data and assuming a base uncertainty of 1.2% in the resistance measurements.

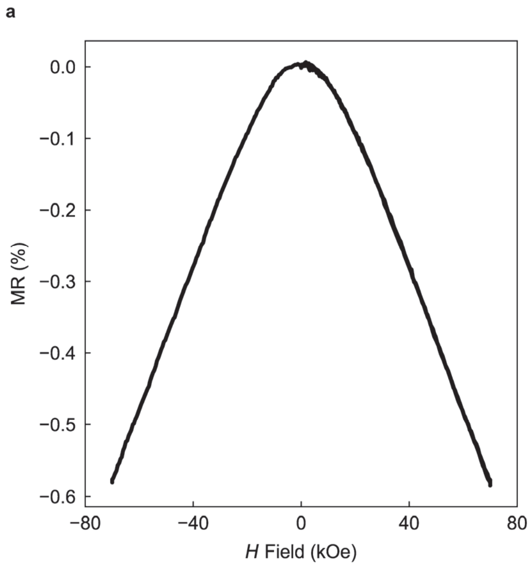

Supplementary Figure 6 Magnetoresistance (MR) of annealed MXenes. MR of Ti_3CNT_x **(a)** and $Mo_2TiC_2T_x$ **(b)** measured at 10 K. The MR measurements were performed within the PPMS and were conducted after the samples had been annealed within the TEM to ≥700 °C. The negative MR of Ti_3CNT_x is similar to that of $Ti_3C_2T_x$[3], and the positive MR of the $Mo_2TiC_2T_x$ sample is consistent with previous reports[4].

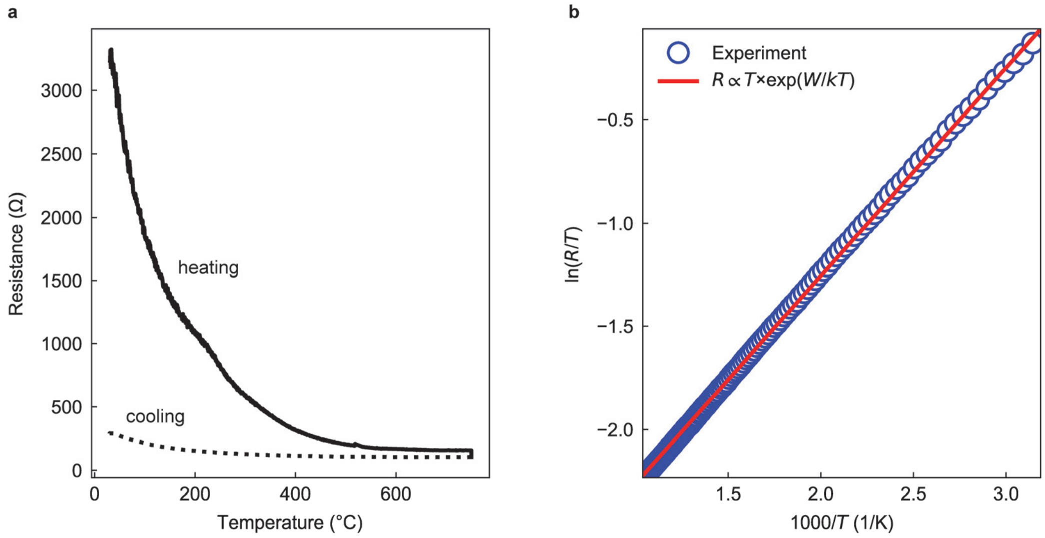

Supplementary Figure 7 Summary of $Ti_3CNT_x(TBA^+)$ heating and biasing results. **a,** Resistance versus temperature for the $Ti_3CNT_x(TBA^+)$ sample during an anneal at 750 °C. The sample was held at 750 °C for 1 h. Prior to this measurement, the sample was held at 200 °C to achieve adequate contact between the MXene film and Pt electrodes, given the presence of free (i.e. non-intercalated) TBA^+, which prevented good electrical contacts. Heating is shown with a solid line and cooling is shown with a dotted line. The large decrease in resistance is mainly attributed to the loss of TBA^+, as seen in Supplementary Figure 4. **b,** Fitting of the cooling curve shown in **a**. The sample closely follows the relation $R \propto T \times \exp(W/kT)$, with W ~ 80 meV and an R value of 0.999. The physical meaning behind this relation is unclear, but we speculate that it is related to the inter-flake hopping mechanism.

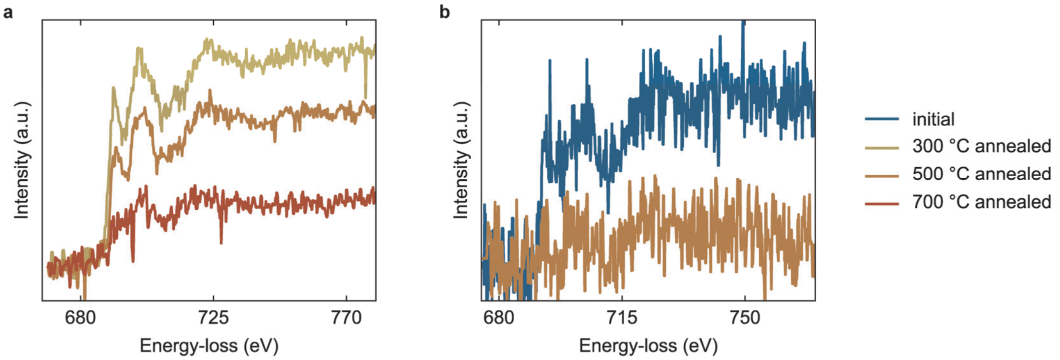

Supplementary Figure 8 Supplementary EELS data. **a,** *In situ* EELS measurements of the Ti_3CNT_x F *K*-edge demonstrating the reduction in –F concentration with vacuum annealing. **b,** *In situ* EELS measurements of the $Mo_2TiC_2T_x$ F *K*-edge demonstrating the elimination of –F terminations with annealing at 500 °C. EELS data was acquired at room temperature after annealing.

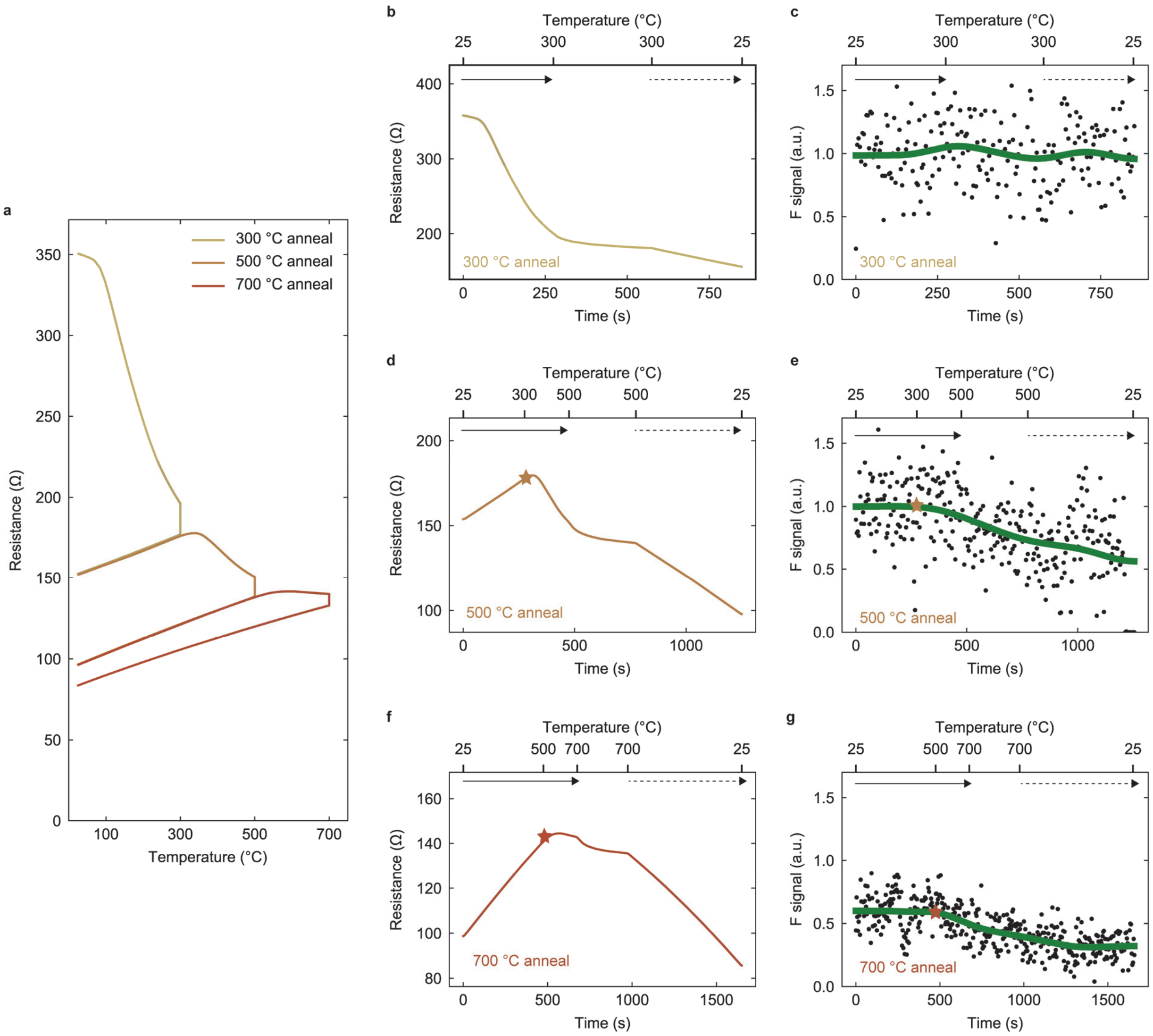

Supplementary Figure 9 Time-resolved EELS and biasing of Ti_3CNT_x. **a,** Change in resistance of Ti_3CNT_x (a different sample from the one described in Figs. 1 and 2 of the main text) with *in situ* annealing. The first annealing step for this sample was at 300 °C, and during this annealing step, there was a transition from negative to positive *dR*/*dT*. The sample was then annealed at 500 and 700 °C. **b, d,** and **f,** show the time-resolved changes in resistance for each annealing step. Solid (dashed) arrows represent sample heating (cooling). For comparison, **c, e,** and **g** show the time-resolved normalized intensity of the F *K*-edge, measured with *in situ* EELS. Each individual spectrum, shown with black dots, had an exposure time of 4 seconds. The green line is a smooth of the data. The stars in **d-g** show the maximum temperature of the prior annealing step. A clear correlation is observed between the onset of –F termination loss and the decrease in MXene resistance.

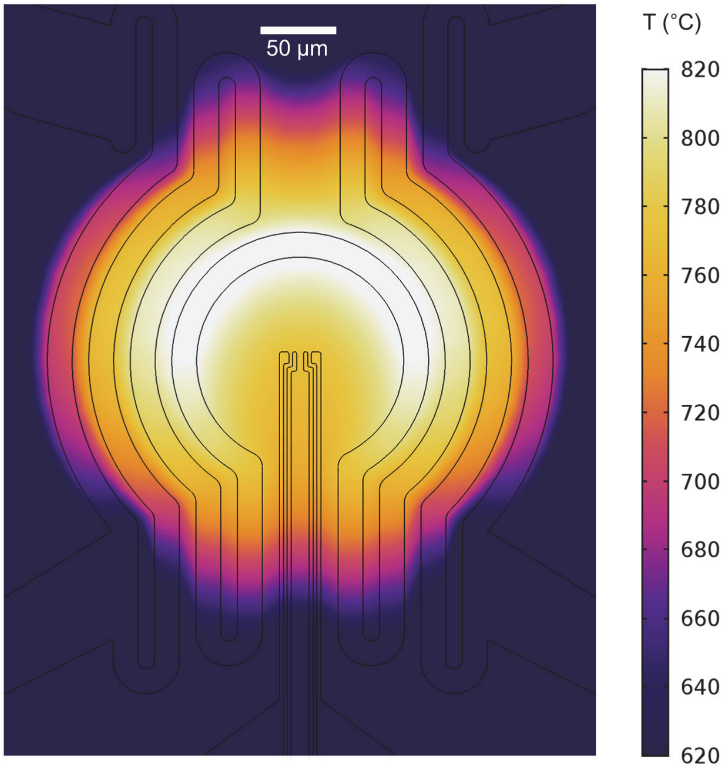

Supplementary Figure 10 Thermal gradients in nanochip. Simulation showing thermal gradients within the nanochip when the set temperature is 775 °C.

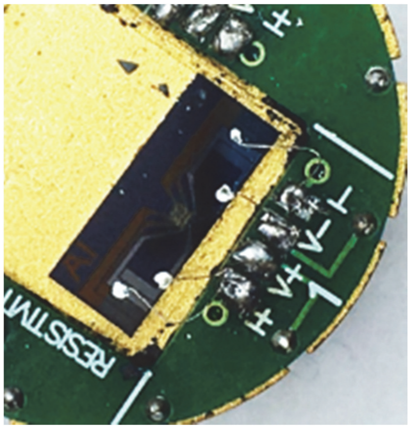

Supplementary Figure 11 PPMS device set-up. Optical photograph of the heating and biasing nanochip connected to the PPMS sample puck.

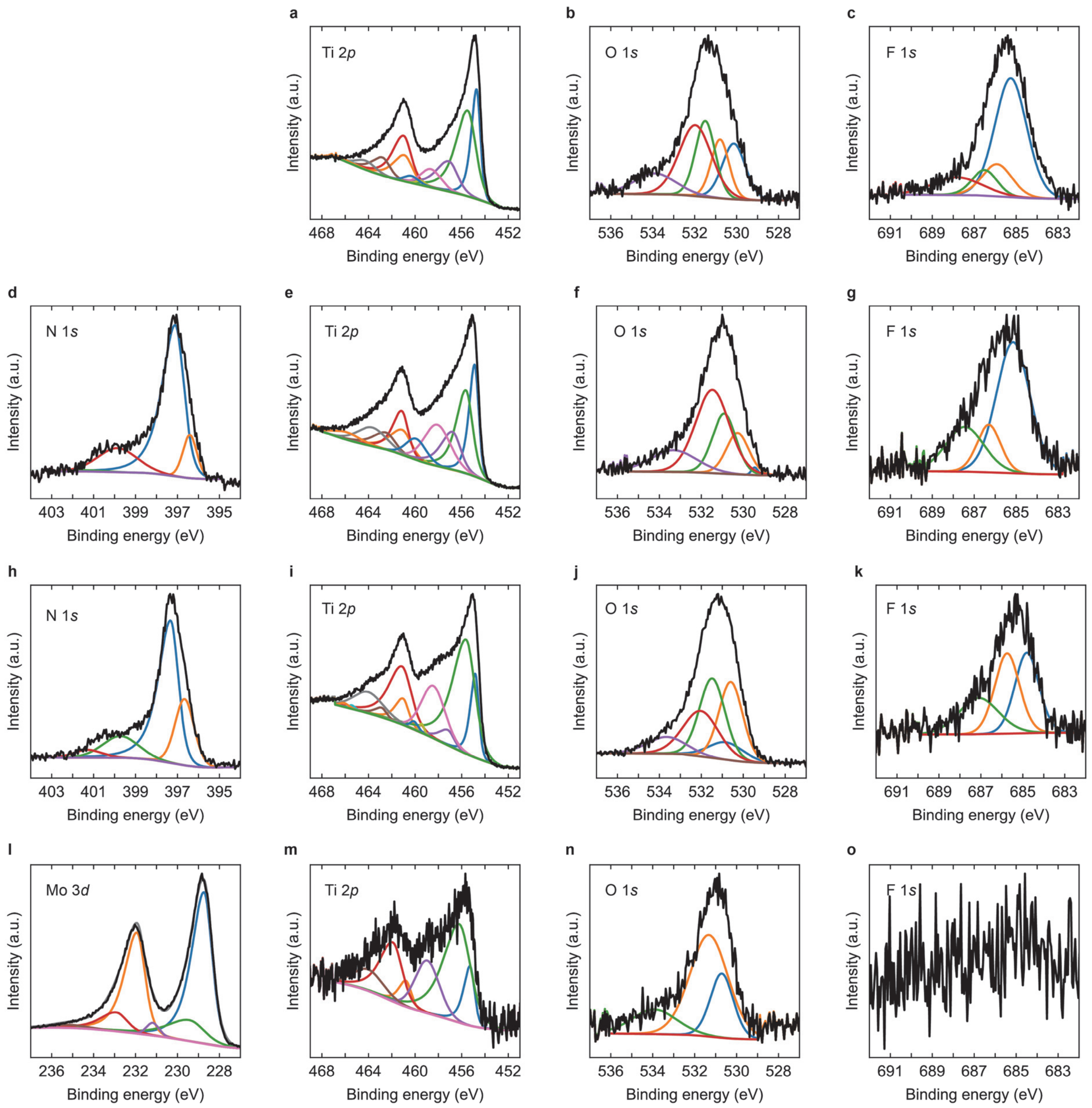

Supplementary Figure 12 XPS data of all as-prepared MXenes. Results for $Ti_3C_2T_x$ **(a-c)**, Ti_3CNT_x **(d-g)**, $Ti_3CNT_x(TBA^+)$ **(h-k)**, and $Mo_2TiC_2T_x$ **(l-o)** are shown.

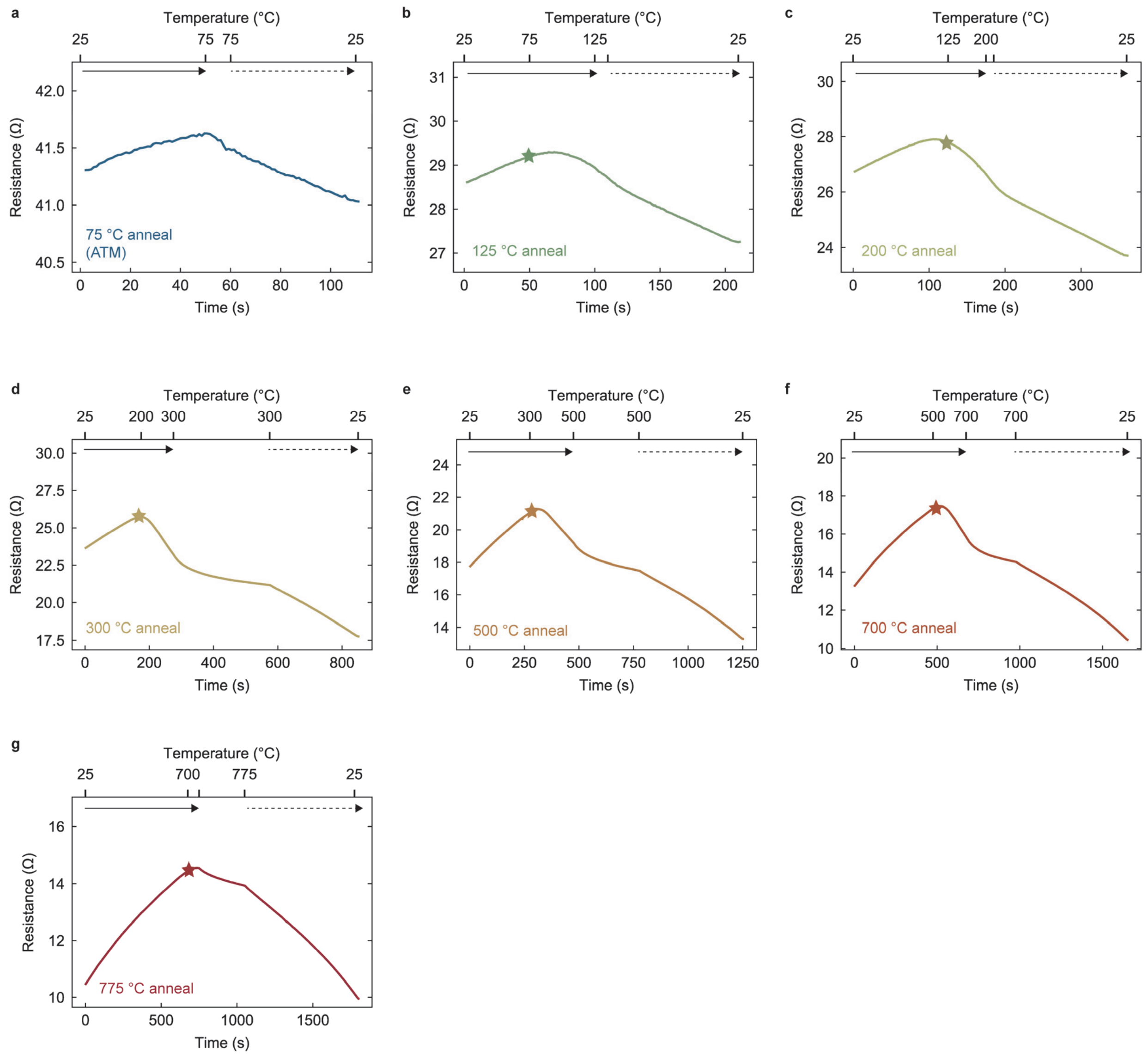

Supplementary Figure 13 Time resolved resistance measurements of $Ti_3C_2T_x$. The data is the same as that presented within Fig. 1 of the main text. Heating and cooling rates were 1 °C/s. Solid (dashed) arrows represent sample heating (cooling). For **a,** the sample was annealed in atmosphere, and for **b-g,** the sample was annealed within the TEM. In between heating and cooling, the sample was held at the annealing temperature for 10 s for **a-c**, and 5 min for **d-g**. In addition to labeling the initial temperature (25 °C) and the maximum annealing temperature, we also mark the temperature where the previous annealing step was performed (marked with a star). In some cases, not all tick marks on the top x-axis (the temperature axis) could be labeled. The unmarked ticks in **b**, **c**, and **g**, correspond to 125, 200, and 775 °C, respectively.

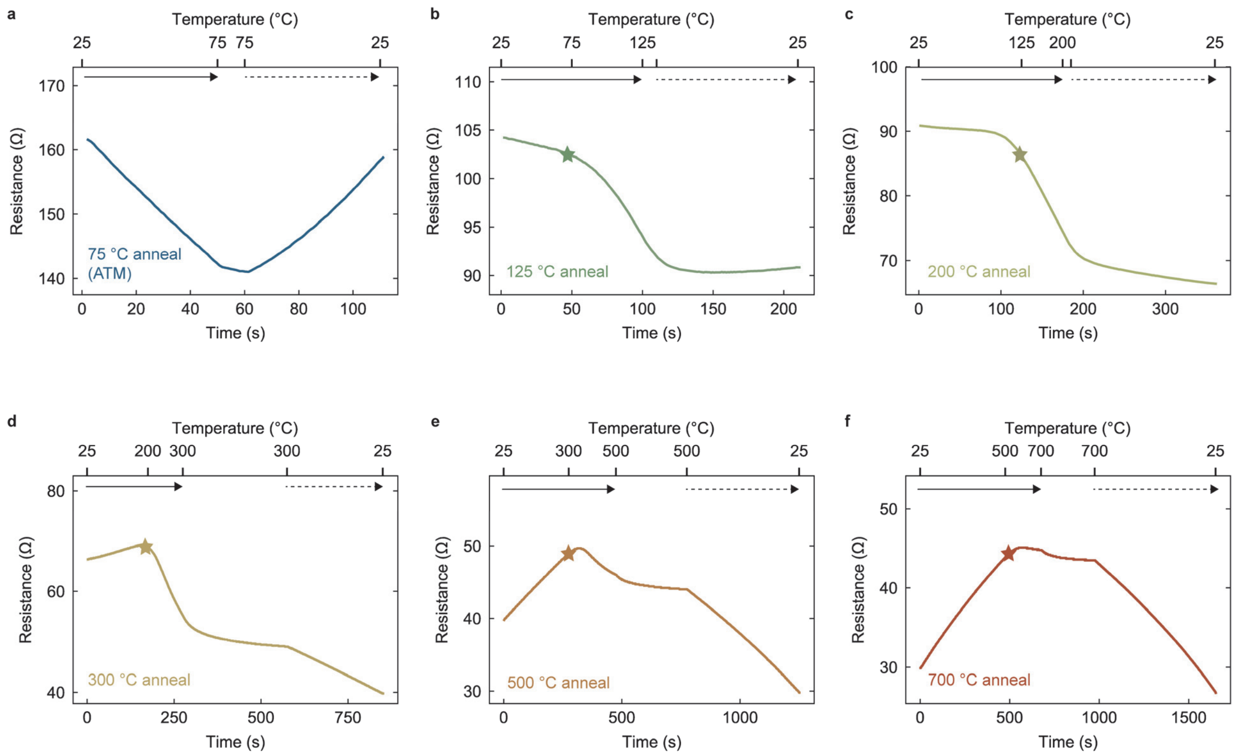

Supplementary Figure 14 Time resolved resistance measurements of Ti_3CNT_x. The data is the same as that presented within Fig. 1 of the main text. Heating and cooling rates were 1 °C/s. Solid (dashed) arrows represent sample heating (cooling). For **a,** the sample was annealed in atmosphere, and for **b-f,** the sample was annealed within the TEM. In between heating and cooling, the sample was held at the annealing temperature for 10 s for **a-c**, and 5 min for **d-f**. In addition to labeling the initial temperature (25 °C) and annealing temperature, we also mark the temperature where the previous annealing step was performed (marked with a star). In some cases, not all tick marks on the top x-axis (the temperature axis) could be labeled. The unmarked ticks in **b** and **c** correspond to 125 and 200 °C, respectively.

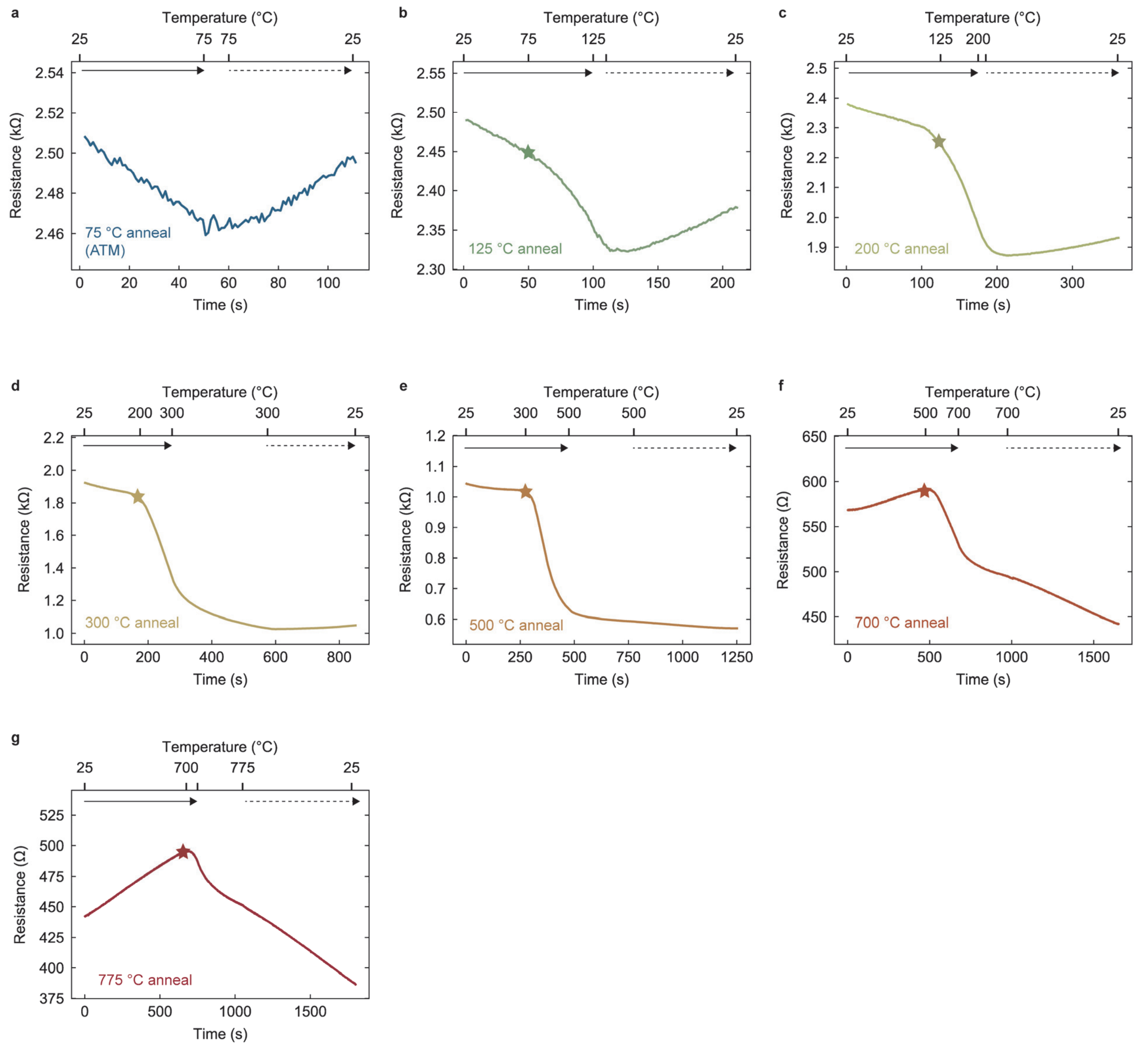

Supplementary Figure 15 Time resolved resistance measurements of $Mo_2TiC_2T_x$. The data is the same as that presented within Fig. 1 of the main text. Heating and cooling rates were 1 °C/s. Solid (dashed) arrows represent sample heating (cooling). For **a,** the sample was annealed in atmosphere, and for **b-g,** the sample was annealed within the TEM. In between heating and cooling, the sample was held at the annealing temperature for 10 s for **a-c**, and 5 min for **d-g.** In addition to labeling the initial temperature (25 °C) and annealing temperature, we also mark the temperature where the previous annealing step was performed (marked with a star). In some cases, not all tick marks on the top x-axis (the temperature axis) could be labeled. The unmarked ticks in **b**, **c**, and **g**, correspond to 125, 200, and 775 °C, respectively.

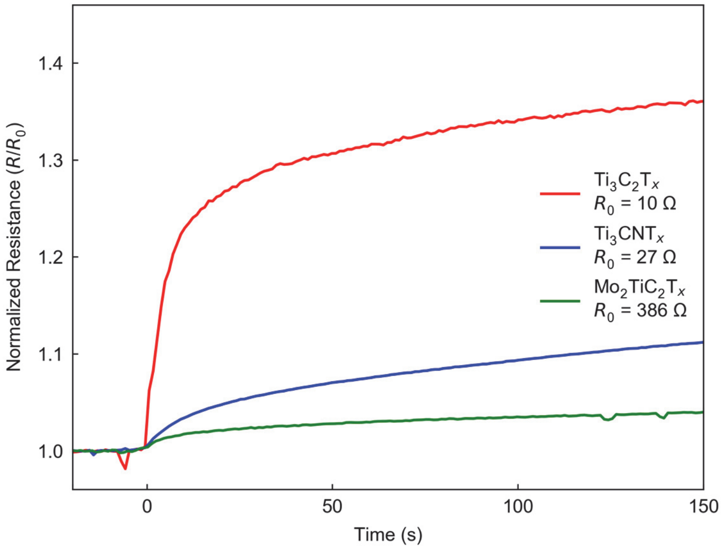

Supplementary Figure 16 Effect of molecular adsorption and surface re-functionalization. Normalized resistance of the $Ti_3C_2T_x$, Ti_3CNT_x, and $Mo_2TiC_2T_x$ MXene films measured as they were removed from the TEM. R_0 is the resistance at time = 0 s, right before the sample was removed from the TEM vacuum and exposed to atmosphere. The increase in resistance for all samples is attributed to molecular absorption and surface re-functionalization.

Supplementary Tables

Supplementary Table 1 Changes in *R* and *dR*/*dT* with *in situ* annealing

Annealing *T* (°C)	$Ti_3C_2T_x$		Ti_3CNT_x		$Ti_3CNT_x(TBA^+)$		$Mo_2TiC_2T_x$	
	R (Ω)	*d*R/*d*T	*R* (Ω)	*d*R/*d*T	*R* (Ω)	*d*R/*d*T	*R* (Ω)	*d*R/*d*T
As-prepared	41.0	.009	159	-.437	-	-	2500	-.84
125	27.25	.0134	90.84	-.0116	-	-	2378.5	-.82
200	23.70	.0139	66.41	.0178	3330	-21.2	1931.3	-.59
300	17.76	.0140	39.80	.0350	-	-	1047.9	-.17
500	13.31	.0110	29.83	.0345	-	-	570.7	.009
700	10.46	.0081	26.87	.0308	-	-	442.1	.069
750	-	-	-	-	290	-1.16	-	-
775	9.95	.0076	-	-	-	-	386.6	.080
Atm	15.14	.011	31.85	.035	-	-	409.6	.0914

Table footnote: The room temperature resistance and *dR*/*dT* values are given for each sample after annealing at various temperatures. The as-prepared data was acquired in ambient atmosphere, and all other annealing steps were performed within the TEM.

Supplementary Table 2 Summary of *in situ* heating and biasing results not included in the manuscript

MXene Chemistry	As-prepared		Max Annealing *T*	Annealed				
	Intercalant	*dR*/*dT*	°C	F	O	Intercalant	*dR*/*dT*	R_I/R_A
$Ti_3C_2T_x$	H_2O, Li^+	+	790	0.5	0.9	Li^+	+	1.5
Ti_3CNT_x	H_2O, Li^+	–	700	0.3	0.9	Li^+	+	4.5
$Ti_3CNT_x(TBA^+)$	H_2O, TBA^+	–	700	-	-	-	–	>10
$Mo_2TiC_2T_x$	H_2O, TBA^+	–	790	0	0.6	-	+	8

Table footnote: The F and O columns give the intensity of fluorine and oxygen *K*-edges after the final annealing step, normalized to the initial edge intensity, i.e. a value of 1 means no change in intensity, and a value of 0 means the complete disappearance of the edge. R_I/R_A gives the ratio of the initial to the final resistance. For these samples, the resistance was not measured prior to insertion into the TEM, so changes in resistance do not reflect the desorption of adsorbed molecules. The initial resistance was measured within the TEM. The $Ti_3CNT_x(TBA^+)$ sample was too thick for EELS analysis.

Supplementary Table 3 XPS of $Ti_3C_2T_x$

Region	BE (eV)	FWHM (eV)	Fraction (%)	Assigned to
Ti $2p_{3/2}$ ($2p_{1/2}$)	454.7 (460.9)	0.8 (1.6)	26.2	C-Ti-T_x
	455.5 (460.1)	1.6 (1.6)	44.8	C-Ti^{2+}-T_x
	457.1 (462.8)	1.8 (1.5)	16.9	C-Ti^{3+}-T_x
	458.7 (464.3)	2.0 (2.0)	8.7	TiO_2
	459.5 (465.5)	0.9 (0.9)	1.0	TiO_2-F
	460.3 (466.3)	1.4 (3.0)	2.4	C-Ti-F
O 1*s*	530.1	1.3	18.4	TiO_2
	530.8	1.0	15.1	TiO_2-F_x
	531.5	1.1	21.4	C-Ti-O_x
	532.0	1.	32.7	C-Ti-OH_x
	533.9	2.4	12.4	H_2O ads
F 1*s*	685.3	1.8	60.2	C-Ti-F_x
	685.9	1.7	15.4	TiO_2-F_x
	686.5	1.4	10.4	AlF_x
	687.8	2.8	14.0	$Al(OF)_x$

Table footnote: The corresponding raw data is shown in Supplementary Figure 12. The data was fit as in ref. 5. The chemical formula was determined to be $Ti_3C_{1.93}O_{0.46}OH_{0.44}F_{0.37}$.

Supplementary Table 4 XPS of Ti_3CNT_x

Region	BE (eV)	FWHM (eV)	Fraction (%)	Assigned to
Ti $2p_{3/2}$ ($2p_{1/2}$)	454.9 (461.1)	0.8 (1.8)	23.3	C-Ti-T_x
	455.6 (461.3)	1.3 (1.3)	30.0	C-Ti^{2+}-T_x
	456.8 (462.5)	1.6 (1.8)	16.3	C-Ti^{3+}-T_x
	458.1 (463.7)	2.6 (2.9)	21.6	TiO_2
	459.1 (465.1)	0.2 (0.7)	0.3	TiO_2-F
	459.9 (465.9)	2.0 (2.8)	8.5	C-Ti-F
O 1*s*	529.5	0.4	0.8	TiO_2
	530.3	1.3	15.2	TiO_2-F_x
	531.0	1.3	22.8	C-Ti-O_x
	531.5	1.9	45.2	C-Ti-OH_x
	533.4	2.6	16.1	H_2O ads
F 1*s*	685.2	1.9	60.7	C-Ti-F_x
	686.3	1.3	15.6	TiO_2-F_x
	686.7	2.1	23.7	AlF_x
N 1*s*	396.4	0.8	11.5	TiCN
	397.1	1.0	69.8	N-Ti-T_x
	399.9	2.2	18.0	NO_x
	401.5	1.6	0.7	NH_4^+

Table footnote: The corresponding raw data is shown in Supplementary Figure 12. The data was fit as in ref. 5. The chemical formula was determined to be $Ti_3C_{1.42}N_{0.59}O_{0.69}OH_{0.88}F_{0.47}$.

Supplementary Table 5 XPS of $Ti_3CNT_x(TBA^+)$

Region	BE (eV)	FWHM (eV)	Fraction (%)	Assigned to
Ti $2p_{3/2}$ ($2p_{1/2}$)	454.8 (461.0)	0.7 (1.2)	15.1	C-Ti-T_x
	455.6 (461.3)	1.8 (1.9)	54.6	C-Ti^{2+}-T_x
	457.2 (462.9)	1.2 (1.2)	4.8	C-Ti^{3+}-T_x
	458.4 (463.0)	2.4 (3.0)	23.5	TiO_2
	459.4 (465.4)	0.5 (0.6)	0.8	TiO_2-F
	460.1 (466.1)	0.8 (0.8)	1.2	C-Ti-F
O 1*s*	530.5	1.3	27.4	TiO_2
	530.8	1.9	9.1	TiO_2-F_x
	531.4	1.4	30.0	C-Ti-O_x
	532.0	2.0	23.9	C-Ti-OH_x
	533.6	2.2	9.6	H_2O ads
F 1*s*	684.9	1.3	36.1	C-Ti-F_x
	685.8	1.4	37.0	TiO_2-F_x
	687.1	2.2	26.9	AlF_x
N 1*s*	396.7	1.0	23.2	TiCN
	397.3	0.9	58.5	N-Ti-T_x
	399.7	2.0	14.2	NO_x, $N(CH_3)_3$
	401.3	1.5	4.0	$N(CH_3)_4^+$

Table footnote: The corresponding raw data is shown in Supplementary Figure 12. The data was fit as in ref. 5. The chemical formula was determined to be $Ti_3C_{0.93}N_{0.67}O_{1.16}OH_{0.55}F_{0.1}$

Supplementary Table 6 XPS of $Mo_2TiC_2T_x$

Region	BE (eV)	FWHM (eV)	Fraction (%)	Assigned to
Ti $2p_{3/2}$ ($2p_{1/2}$)	455.3 (460.8)	0.9 (1.8)	14.4	C-Ti^{2+}
	456.2 (461.9)	2.0 (2.1)	60.1	C-Ti^{3+}
	458.9 (464.0)	2.5 (3.0)	25.5	TiO_2
Mo $3d_{5/2}$ ($3d_{3/2}$)	228.7 (231-9)	0.9 (0.9)	77.5	C-Mo-T_x
	229.4 (232.9)	1.3 (1.7)	19.0	Mo satellite
	231.2 (235.2)	0.7 (1.9)	3.5	MoO_3
O 1*s*	530.7	1.3	22.9	MoO_3
	531.3	2.2	60.0	C-Mo-O_x
	533.9	2.5	17.1	C-Mo-OH_x

Table footnote: The corresponding raw data is shown in Supplementary Figure 12. The data was fit as in ref. 5. The chemical formula was determined to be $Mo_{2.11}TiC_{2.1}O_{1.56}OH_{0.45}F_{0.01}$

Supplementary Note 1

MXene *dR*/*dT* analysis: To better understand the effects of de-intercalation and surface de-functionalization on MXene electronic properties, we analyze the changes in MXene *dR*/*dT* with *in situ* annealing. We argued in the main text that $Ti_3C_2T_x$, Ti_3CNT_x and $Mo_2TiC_2T_x$ are all intrinsically metallic in their as-prepared state, and that the semiconductor-like behavior of Ti_3CNT_x and $Mo_2TiC_2T_x$ is due to inter-flake effects. This argument implies that the ensemble resistance of each MXene studied here, across all annealing temperatures, can be described by a metallic intra-flake term in series with an insulating inter-flake term

$$R \propto \frac{m\omega(T)}{e^2 n} + R_0 \exp\left(\frac{T_0}{T}\right)^p \qquad (1)$$

The equation is a proportional equation because the resistance is also dependent upon the device geometry. The first term of Supplementary Equation 1 is the metallic (Drude) intra-flake resistance, characterized by the effective electron mass, *m*, the electron scattering rate, *ω*, and the carrier density, *n*. This metallic term contributes a positive component to the ensemble value of *dR*/*dT* owing to the linear temperature dependence of *ω* at high temperatures due to electron-phonon coupling[6,7]. The second term represents the insulating inter-flake resistance, where *p* differentiates between thermally activated transport and variable range hopping formulae; these formulae have previously been used to successfully fit semiconductor-like MXene behavior[4,8,9]. The inter-flake term contributes a negative component to the ensemble value of *dR*/*dT*. Because the intra-flake and inter-flake resistances have opposing temperature dependencies, the ensemble value of *dR*/*dT* is determined by the balance of these two terms. Owing to this balance, a decrease in the metallic intra-flake resistance of Supplementary Equation 1 (through, e.g., a change in *n* with annealing) will *decrease* the ensemble value of *dR/dT*. Conversely, a decrease in the insulating inter-flake resistance (through a change in R_0 or T_0 with annealing) will *increase*

dR/*dT*. Thus, by measuring the change in *dR*/*dT* with annealing, we can differentiate between a decrease in the inter-flake resistance (mediated by de-intercalation) or a decrease in the intra-flake resistance (driven by termination loss).

To verify the preceding claims regarding changes in *dR*/*dT* and inter-flake versus intra-flake effects, we consider the ensemble value of *dR*/*dT*. For the intra-flake Drude term, the total electron scattering rate, ω, is the sum of many scattering processes. In general, the two largest scattering processes are impurity scattering and electron-phonon scattering[6]. The total resistance is then given by

$$R \propto \left[\frac{m\omega_{\mathrm{i}}}{e^2 n} + \frac{m\omega_{\mathrm{p}}(T)}{e^2 n}\right] + R_0 \exp\left(\frac{T_0}{T}\right)^p \tag{2}$$

where the brackets encompass the metallic intra-flake resistance, ω_{i} is the impurity scattering rate, and ω_{p} is the electron-phonon scattering rate. Impurity scattering is largely temperature independent, but electron-phonon scattering is temperature dependent. Above the Debye temperature, ω_{p} scales linearly in temperature[6,7]. For our *in situ* TEM measurements from RT up to 775 °C, we are in the regime where ω_{p} is proportional to *T*. This claim is supported by the approximately constant temperature dependence of resistance measured for $Ti_3C_2T_x$, Ti_3CNT_x, and $Mo_2TiC_2T_x$ after high temperature annealing (Fig. 1). As such, the ensemble value of *dR*/*dT* is given by

$$\frac{dR}{dT} \propto \frac{mC_{\mathrm{p}}}{e^2 n} - \frac{pR_0\left(\frac{T_0}{T}\right)^p exp\left(\frac{T_0}{T}\right)^p}{T} \tag{3}$$

where C_{p} is the temperature coefficient of electron-phonon scattering, i.e. $\omega_{\mathrm{p}} = TC_{\mathrm{p}}$. Our aim is to understanding how the ensemble value of *dR*/*dT* changes given a change in either the intra-flake or the inter-flake resistance terms. To understand this correlated behavior, we take the

derivatives of R and dR/dT with respect to the material parameters which determine the ensemble resistance, i.e. ω_i, m, n, C_p, R_0, and T_0. First we investigate the intra-flake term:

$$\frac{dR}{d\omega_i} \propto \frac{m}{e^2 n} \tag{4}$$

$$\frac{\partial^2 R}{\partial \omega_i \partial T} = 0 \tag{5}$$

$$\frac{dR}{dm} \propto \frac{\omega_i + C_p T}{e^2 n} \tag{6}$$

$$\frac{\partial^2 R}{\partial m \partial T} \propto \frac{C_p}{e^2 n} \tag{7}$$

$$\frac{dR}{dC_p} \propto \frac{mT}{e^2 n} \tag{8}$$

$$\frac{\partial^2 R}{\partial C_p \partial T} \propto \frac{m}{e^2 n} \tag{9}$$

$$\frac{dR}{dn} \propto -\frac{m(\omega_i + C_p T)}{e^2 n^2} \tag{10}$$

$$\frac{\partial^2 R}{\partial n \partial T} \propto -\frac{m C_p}{e^2 n^2} \tag{11}$$

The main takeaway from Supplementary Equations 4-11 is that a decrease in R driven by a change in m, C_p, or n will be accompanied by a decrease in dR/dT. For example, dR/dm and $\partial^2 R/\partial m \partial T$ are both positive, hence, an increase in m will increase both R and dR/dT. For ω_i, $\partial^2 R/\partial \omega_i \partial T$ is zero, thus a change in resistance driven by a change in ω_i will not affect the ensemble value of dR/dT.

Next we consider the insulating inter-flake term:

$$\frac{dR}{dR_0} \propto \exp\left(\frac{T_0}{T}\right)^p \tag{12}$$

$$\frac{\partial^2 R}{\partial R_0 \partial T} \propto -\frac{p\left(\frac{T_0}{T}\right)^p exp\left(\frac{T_0}{T}\right)^p}{T} \tag{13}$$

$$\frac{dR}{dT_0} \propto \frac{pR_0\left(\frac{T_0}{T}\right)^p exp\left(\frac{T_0}{T}\right)^p}{T_0} \tag{14}$$

$$\frac{\partial^2 R}{\partial T_0 \partial T} \propto -\frac{p^2 R_0\left(\frac{T_0}{T}\right)^p exp\left(\frac{T_0}{T}\right)^p\left(\left(\frac{T_0}{T}\right)^p+1\right)}{T_0 T} \tag{15}$$

The derivative of R with respect to R_0 and T_0 is positive, but the derivative of dR/dT with respect to R_0 and T_0 is negative. Hence, a decrease in resistance driven by a change in R_0 or T_0 will increase the ensemble value of dR/dT. We assume that the inter-flake transport mechanism does not change with annealing, i.e. the exponent p does not change.

Next, we consider the specific case where the insulating inter-flake resistance is negligible, and with annealing, the intra-flake resistance decreases solely due to a change in n

$$R \propto \frac{1}{n}\left(\frac{m\omega_i}{e^2} + \frac{m\omega_{\mathrm{p}}(T)}{e^2}\right) \tag{16}$$

$$\frac{dR}{dT} \propto \frac{1}{n}\left(\frac{mC_{\mathrm{p}}}{e^2}\right) \tag{17}$$

We calculate the proportional change in R given a change in n with annealing, where the proportional change in resistance is defined as the resistance after annealing, R_2, minus the initial resistance, R_1, divided by the initial resistance

$$\frac{R_2-R_1}{R_1} = \frac{\left[\frac{1}{n_2}\left(\frac{m\omega_{\mathrm{i}}}{e^2}+\frac{m\omega_{\mathrm{p}}(T)}{e^2}\right)\right]-\left[\frac{1}{n_1}\left(\frac{m\omega_{\mathrm{i}}}{e^2}+\frac{m\omega_{\mathrm{p}}(T)}{e^2}\right)\right]}{\left[\frac{1}{n_1}\left(\frac{m\omega_{\mathrm{i}}}{e^2}+\frac{m\omega_{\mathrm{p}}(T)}{e^2}\right)\right]} = \frac{\frac{1}{n_2}-\frac{1}{n_1}}{\frac{1}{n_1}} = \frac{n_1}{n_2} - 1 \tag{19}$$

where n_1 is the initial carrier concentration and n_2 is the carrier concentration after annealing. Similarly, we calculate the proportional change in dR/dT given a change in n with annealing

$$\frac{\frac{dR}{dT_2}-\frac{dR}{dT_1}}{\frac{dR}{dT_1}} = \frac{\left[\frac{1}{n_2}\left(\frac{mC_{\mathrm{p}}}{e^2}\right)\right]-\left[\frac{1}{n_1}\left(\frac{mC_{\mathrm{p}}}{e^2}\right)\right]}{\left[\frac{1}{n_1}\left(\frac{mC_{\mathrm{p}}}{e^2}\right)\right]} = \frac{\frac{1}{n_2}-\frac{1}{n_1}}{\frac{1}{n_1}} = \frac{n_1}{n_2} - 1 \tag{20}$$

Importantly, the proportional change in dR/dT with a change in n is equivalent to the proportional change in R.

To represent how the MXene resistance and dR/dT evolve with annealing, we introduce a parameter η, defined as the ratio of the proportional change in the RT dR/dT to the proportional change in the RT resistance for a given annealing step

$$\eta = \frac{\frac{\frac{dR}{dT_2} - \frac{dR}{dT_1}}{\mathrm{abs}\left(\frac{dR}{dT_1}\right)}}{\frac{R_2 - R_1}{R_1}} \quad (21)$$

where abs() denotes the absolute value. If the change in resistance with annealing is solely due to a change in the intra-flake carrier concentration (and the relative value of the inter-flake resistance is negligible) then the Drude equation predicts η = 1. More generally, if both the resistance and dR/dT decrease during an annealing step, then η is positive (consistent with a change to the intra-flake resistance). If the resistance decreases and the value of dR/dT increases during an annealing step, then η is negative (consistent with a change to the inter-flake resistance). This formulation of η also allows the analysis of increases in resistance, for example, when the MXenes are removed from the TEM and exposed to ambient atmosphere (Supplementary Figure 16).

Supplementary Figure 5 plots η for each MXene for each *in situ* annealing step. For annealing at 125 and 200 °C, η is negative for all MXenes, consistent with a decrease in the inter-flake resistance driven by de-intercalation of H_2O. At higher annealing temperatures, η tends towards positive values for $Ti_3C_2T_x$ and Ti_3CNT_x, consistent with a transition from de-intercalation to surface de-functionalization control of resistance. For $Ti_3C_2T_x$, η ~ 1 for annealing at 500, 700,

and 775 °C, and for Ti_3CNT_x, $\eta \sim 1$ for annealing at 700 °C. This data is consistent with an increase in the MXene intra-flake conductivity *via* surface de-functionalization and an increase in n. The slower transition from negative to positive η for Ti_3CNT_x relative to $Ti_3C_2T_x$ is indicative of the increased lattice response of Ti_3CNT_x to H_2O intercalation, as evidenced by XRD measurements (Fig. 2c). For $Mo_2TiC_2T_x$, η remains negative for every *in situ* annealing step. This behavior suggests that at all annealing temperatures, the measured decreases in $Mo_2TiC_2T_x$ resistance are due (in part) to decreases in the inter-flake resistance. We speculate that residue from the TBA^+ decomposition continues to affect the inter-flake resistance for annealing at ≥775 °C, causing $\eta < 0$. This claim is supported by the $Ti_3CNT_x(TBA^+)$ sample, where $\eta < 0$ for annealing at 500 and 700 °C, in contrast to Ti_3CNT_x intercalated with only H_2O and Li^+. The negative value of η for $Mo_2TiC_2T_x$ does not, however, contradict a correlation between –O termination loss and improved conductivity in $Mo_2TiC_2T_x$. It is also possible that the negative value of η for $Mo_2TiC_2T_x$ arises due to a nontrivial relation between surface termination loss and intra-flake conductivity, which is not captured by the simple Drude formula. We also calculated η as the MXenes were removed from the TEM and exposed to ambient atmosphere (Supplementary Figure 5 and Supplementary Figure 16). For all three MXenes, this process caused an increase in resistance and an increase in dR/dT, giving $\eta \sim 1$. As such, we attribute this increase in resistance to a decrease in carrier concentration with surface re-functionalization and molecular adsorption.

Supplementary References

1. Sang, X. *et al.* In situ atomistic insight into the growth mechanisms of single layer 2D transition metal carbides. *Nat. Commun.* **9,** 1–9 (2018).

2. Ghassemi, H. *et al.* In situ environmental transmission electron microscopy study of oxidation of two-dimensional Ti_3C_2 and formation of carbond-supported TiO_2. *J. Mater. Chem. C* **2,** 14339–14343 (2014).

3. Halim, J. *et al.* Transparent Conductive Two-Dimensional Titanium Carbide Epitaxial Thin Films. *Chem. Mater.* **26,** 2374–2381 (2014).

4. Anasori, B. *et al.* Control of electronic properties of 2D carbides (MXenes) by manipulating their transition metal layers. *Nanoscale Horizons* **1,** 227–234 (2016).

5. Halim, J. *et al.* Applied Surface Science X-ray photoelectron spectroscopy of select multi-layered transition metal carbides (MXenes). *Appl. Surf. Sci.* **362,** 406–417 (2016).

6. Kasap, S. O. *Principles of Electronic Materials and Devices*. (McGrow-Hill, 2006).

7. Kittel, C. *Introduction to Solid State Physics*. (John Wiley & Sons, 2005).

8. Halim, J. *et al.* Variable range hopping and thermally activated transport in molybdenum-based MXenes. *Phys. Rev. B* **98,** 104202 (2018).

9. Halim, J. *et al.* Synthesis and Characterization of 2D Molybdenum Carbide (MXene). *Adv. Funct. Mater.* **26,** 3118–3127 (2016).
